# Health-related quality of life in undergraduate women using any contraceptive

**DOI:** 10.1186/s12955-019-1157-2

**Published:** 2019-05-24

**Authors:** Fatima Leon-Larios, Cinta G Vazquez-Valeo, Alicia Sanchez-Sanchez, Diego Gomez-Baya, Juana Macías-Seda, M Nieves Cabezas-Palacios

**Affiliations:** 1 Nursing Department, Faculty of Nursing, Physiotherapy and Podiatry, University of Seville, Seville, Spain; 2 Hospital Universitario Juan Ramon Jimenez, Huelva, Spain; 3 Hospital de Merida, Merida, Spain; 4 Department of Social, Developmental and Educational Psychology, University of Huelva, Huelva, Spain; 5 Hospital Universitario Virgen Macarena, Seville, Spain; 6 Department of Social, Developmental and Educational Psychology, Faculty of Education, Psychology and Sport Sciences, Universidad de Huelva, Campus “El Carmen”, Avda. Fuerzas Armadas s/n., 21007 Huelva, Spain

## Abstract

**Objective:**

The aim of this research was to assess university students’ health-related quality of life whether they use some contraceptive method or not.

**Methods:**

This research is a cross-sectional study. Female participants who studied a degree in health at the University of Seville (Spain) were recruited. Respondents completed a demographic questionnaire and health-related quality of life was measured with validated instrument SEC-QoL (Sociedad Española de Contracepción- Quality of Life) in Spanish that measured five dimensions: sexual, social, breast, menstrual and psychosocial.

**Results:**

A total of 992 women aged 21.37 (3.6) years old participated in this study. Women who used a contraceptive method reached higher scores at the SEC-QoL questionnaire 47.09 (17.04) and 46.91 (18.73) than those that didn’t. Likewise, women who chose a hormonal method showed a better overall health-related quality of life, compared to those who used a non-hormonal method. Participants who used hormonal contraceptives obtained higher scores in all health-related quality of life domains (social, menstrual, breast and sexual), except psychological domain when compared to those who used a non hormonal method or none. Furthermore, a moderated mediation model showed that the effect of the current contraceptive method on health-related quality of life was partially explained by the moderated mediation of the time using this method, the reason for using it and the existence of a partner.

**Conclusion:**

The usage of hormonal contraceptives increases health-related quality of life in young women. Several variables regarding the experience with contraceptive methods should be considered in order to examine the effect on health-related quality of life in undergraduate women.

## Plain English summary

Contraceptives present benefits for young women beyond avoiding a pregnancy. This research was designed to assess the impact of contraception on health-related quality of life. A cross-sectional study was carried out with a sample of 992 female university students from the University of Seville using SEC-QoL (Sociedad Española de Contracepción- Quality of Life) scale validated by the Spanish Society of Contraception. The use of any contraceptive method improves women’s quality of life measured. Women who used a hormonal contraceptive had higher health-related quality of life scores. The use of hormonal methods improves all domains in young women, except for the psychological domain at SEC-QoL scale. Several variables about the use of contraceptive methods should be considered in order to examine the effect on health-related quality of life.

## Background

Contraceptive methods present benefits besides contraception [1]. Nevertheless, many women and health professionals are not aware of them [2]. Contraceptives were initially developed to plan pregnancies; however; many studies have demonstrated non-contraceptive benefits [3]. Among these benefits, it was found that hormonal contraceptives reduced menstrual blood loss, dysmenorrhoea and premenstrual syndrome (PMS) [4]. Moreover, they reduce the later incidence of endometrial and ovarian cancer and appear to help to protect future fertility by reducing the risk of acute pelvic floor inflammatory disease, endometriosis and uterine fibroids [5].

Premenstrual symptoms are common in reproductive-aged women. PMS refers to the cyclic occurrence of a set of disturbing physical, emotional or behavioral alterations that are of sufficient severity to interfere with interpersonal relations and routine life, affecting quality of life [6]. Epidemiological studies carried out in Spain showed that 73.7% of menstruating women are affected by some premenstrual symptoms during the menstrual cycle [7], including headaches and premenstrual mood changes, such as anxiety, irritability or fatigue, that are present during spontaneous menstrual cycles and during the use of oral contraceptives [8] with or without a hormone free interval. The impact of premenstrual symptoms on the daily life of women depends on the severity of such symptoms [9]. Many symptoms of PMS are associated with poor health-related quality of life [10, 11] affecting mental, emotional and physical domains [11, 12]. Some studies confirm beneficial effects of hormones on mood [13] and physical symptoms of PMS, while others failed to show effectiveness of hormones [14, 15].

Previous research has demonstrated that using hormonal contraceptives can theoretically reduce PMS symptoms in all forms of hormonal contraception [16]. These effects demonstrate that contraceptives should be taken into consideration regarding quality of life, so this has become a great issue in all health systems [17, 18].

There are studies where women’s health in relation with their quality of life has been measured using standard questionnaires such as EuroQol-5D (EQ-5D) [19], SF-36 [20, 21], World Health Organization’s Quality of Life (WHOQOL) [22, 23], Psychological General Well-Being Index (PGWBI) [18, 24] or Quality of life Enjoyment and Satisfaction Questionnaire (Q-LES-Q) [25]. The Spanish Contraception Society developed a survey in Spanish to specifically measure women’s health-related quality of life when using contraceptive methods [26–28]. This questionnaire, called SEC-QoL (Sociedad Española de Contracepción- Quality of Life) is the only one designed for fertile women providing information on five dimensions.

This study tries to assess associations between contraceptive choice and women’s health-related quality of life in a sample of university students.

## Methods

### Study design and participants

We conducted a cross-sectional study among students at the University of Seville from June to October 2015. Participants answered a self-administered questionnaire based on SEC-QoL, sociodemographic variables and questions about the use of contraceptives and sexual habits: a) current contraceptive method being used; b) contraceptive methods used throughout life; c) partner; d) time with a relationship; e) number of sexual partners in the last year; f) frequency of sexual relations; g) age at first sexual relation; h) motivation to use current contraceptive method.

Women were eligible to participate in this study whether they were: a) older than 18 years; b) having menstrual cycle monthly (except women who do free-interval with hormonal contraceptives). Women who reached menopause were excluded.

### Instrument

Quality of life was assessed with the SEC-QoL questionnaire (see Appendix). The questionnaire was created to assess the impact of contraceptive methods on the health-related quality of life of women. They assumed that the effects associated with the menstrual cycle had an influence on health-related quality of life, particularly in women who used hormonal methods [26].

This is a self-administered questionnaire consisting of 19 items and five domains: social (consisting of 5 items), menstrual symptoms (4 items), breast symptoms (3 items), psychological (4 items) and sexual (3 items). Each item is scored from 1 to 5 likert response choices (from “always” to “never” or from “totally agree” to “totally disagree” depending on the statement). The total score range is 0–100.

This questionnaire showed a good internal consistency with an overall Cronbach’s alpha of 0.88 and a value higher than 0.70 in all domains, except for the sexual domain (0.55). The overall intra-class correlation coefficient was 0.82, which was higher than 0.70 in all domains, thus indicating the good test-retest reliability of the questionnaire (25).

Standardization is obtained as follows: (current score- minimum score)/ (maximum score-minimum score) *100.

### Ethics, consent and permissions

The study was approved by the Ethical committee at the University of Seville and written informed consent was obtained from each participant. The study protocol conformed to the ethical guidelines of the 1975 Helsinki declaration.

### Statistical methods

A descriptive analysis of the sociodemographic characteristics was performed in IBM Corp. Released 2013. IBM Statistical Package for the Social Sciences (SPSS) for Windows, Version 21.0. Armonk, NY: IBM Corp. We compared the characteristics of patients who used any contraceptive method to patients without recent contraceptive use and users of a hormonal method and others. Data were expressed as absolute frequencies, average and percentages. The differences in SEC-QoL scores between hormone users and non-users were calculated by analysis of variance and by chi-square test. A *p* value of less than 0.05 was considered as indicating significance for these analyses. 95% confidence intervals were calculated assuming a Poisson distribution.

Finally, some mediation and moderation analyses were performed to further explore the type of relationships among the study variables. These analyses were carried out following the recommendations described by Preacher et al. [29], based on regression analyses. The effects described in this model represent causal assumptions, because there is no causation verification without variable manipulation. Thus, only associations among variables may be concluded. Standardized coefficients were calculated to estimate the effect of one variable (assumed to be the independent variable) on another variable (assumed to be the criterion variable). Process v3.0 macro for SPSS was used [30], by specifically applying the model number 68, developed by Hayes (2017). A total of 1000 bootstrap samples were estimated for bias-corrected bootstrap confidence intervals for specific indirect effects [31]. This model tested the mechanisms which may underline the relationship between the current contraceptive method being used and health-related quality of life. Time using the contraceptive method is expected to partially mediate this effect on health-related quality of life. In turn, the reason for using the contraceptive method and having a partner or not are proposed to moderate such partial mediation. These analyses were sequentially performed before being integrated in the overall moderated mediation model.

## Results

This study was carried out within a cohort of university students. Population included 1007 women aged between 17 and 54 years old who were enrolled in any of the degrees at the Faculty of Nursing, Physiotherapy and Podiatry from the University of Seville (Spain). A total of 992 (98.5%) women aged between 18 and 50 years old answered the questionnaire, two of which did not meet inclusion criteria because of menopause. Sociodemographic characteristics of participants are shown in Table 1.

**Table 1:** Sociodemographic characteristics of study participants

	N	%
Degree		
Nursing	675	68.0
Physiotherapy	163	16.4
Podiatry	148	14.9
Age		
< 20 years	464	47
20–25 years	448	45.3
25–30 years	53	5.4
> 30 years	23	2.3

The age of participants was 21.37 years old (3.60). All women included were studying a degree in health science at university.

### Sexual habits of participants

Participants who had a partner were 67.60% (665) and were engaged in a relationship that lasted an average of 36.27 (32.02) months. The initiation of sexual intercourse among women who answered was at 16.71 (1.78) years old. Most common frequency of sex was once-twice a week 40.62% (403), more than twice a week 21.37% (212); once-twice a month 19.10% (189) and once-twice a year 4.58% (48). Female students that had not had sex before were 9.90% (98).

### Contraceptive habits of participants

Contraceptive use in women who participated in this study is 90.6%. Students who had used a condom at least once in their lives were 84.57% (839); coitus interruptus by 24.39% (242), hormonal contraceptive pills by 42.54% (422), hormonal vaginal ring by 10.18% (101); contraceptive patches by 1% (10) and subdermal implant by 0.10% (1).

The current most widely used contraceptive method is the condom: 47.07% (467), followed by contraceptive pills: 23.28% (231), and 12.2% (115) do not use any method. However, only a 9.90% (98) referred that they had not had sex before, so 2.52% had sex without using any contraceptive method. Timing using the current method is 24 months (p25 (12)-p75 (48)).

Motivation to choose a method is based on security (55.14% (547)), prevention of sexual transmission infection (STI) (14.61% (145)), ease of use (6.35% (63)), price (1.91% (19)), availability (1.71% (17)). Women who chose a method to prevent STI used a condom more often (*p* < 0.001)

### Quality of life scale

Participants´ mean score (SD) was 54.57 (13.26), (95% CI 53.74 to 55.39) at global SEC-QoL score. If we analyze women who use a contraceptive method in comparison to women who do not, mean scores are 47.09 (17.04) and 46.91 (18.73) respectively. If we detail each score depending on the method we find that hormonal methods show higher scores as it is shown in Table 2. Women using hormonal contraceptive methods reported greater SEC-QoL scores than women using non hormonal ones (*p* < 0.001).

**Table 2:** Health-related quality of life by method of contraception

Method	Frequency (n)	Average SEC-QoL scale	standard deviation	95% CI upperlower	
condom	467	44.87	16.32	43.38	46.35
vaginal ring	46	54.09	17.64	48.85	59.33
coitus interruptus	37	43.24	15.68	38.01	48.47
Contraceptivepills	231	50.24	18.02	47.90	52.58
implants	1	75	–	–	–
patches	2	56.57	3.72	23.14	90.01
no method	115	46.91	18.72	43.45	50.37
doublemethod	39	46.99	14.77	42.20	51.78

When observing every single sub-scale, statistically significant differences were seen between women using hormonal contraceptives and women not using them, as Table 3 shows, except for the psychological dimension. We assume that the most affected domain between young women during the menstrual cycle is the breast symptoms one and the least affected one is the social domain. On the other hand, domains with more differences between hormonal method users and non-users are menstrual symptoms and the sexual domain observing higher scores in hormonal contraceptive users.

**Table 3:** Scores in health-related quality of life domains, by the use of hormonal methods or not

Domain	Non-hormonal methods	Hormonal methods	*p*-value	95% CI	
	Mean (SD)	Mean (SD)		Upper	Lower
Social^a^	15.25 (4.92)	16.03 (4.77)	0.020	−1.44	−0.12
Menstrual^b^	10.93 (3.70)	12.12 (3.87)	0.000	−1.69	−0.68
breast^c^	7.00 (3.45)	7.52 (3.40)	0.027	−0.99	−0.06
Psychological^b^	11.85 (3.77)	12.23 (3.72)	0.135	−0.89	−0.12
Sexual^c^	8.42 (2.77)	9.46 (2.61)	0.000	−1.40	−0.67

^a^25 points maximum in scale; ^b^20 points maximum in scale, ^c^15 points maximum in scale

Health-related quality of life by age did not show any differences among groups under and over 30 years old (46.85 (17.12) vs 47.88 (27.72), *p* = 0.781, 95% CI (− 8.24, 6.19). Time using a contraceptive method is not related to higher SEC-QoL scale scores (*p* > 0.05). In bivariate analysis women who have been using hormonal contraceptives longer did not show better scores (*p* > 0.05). However, significant differences were found between women using hormonal contraceptives who had sex more frequently than women who did not (*p* < 0.001).

### Moderated mediation model

**Fig. 1:**
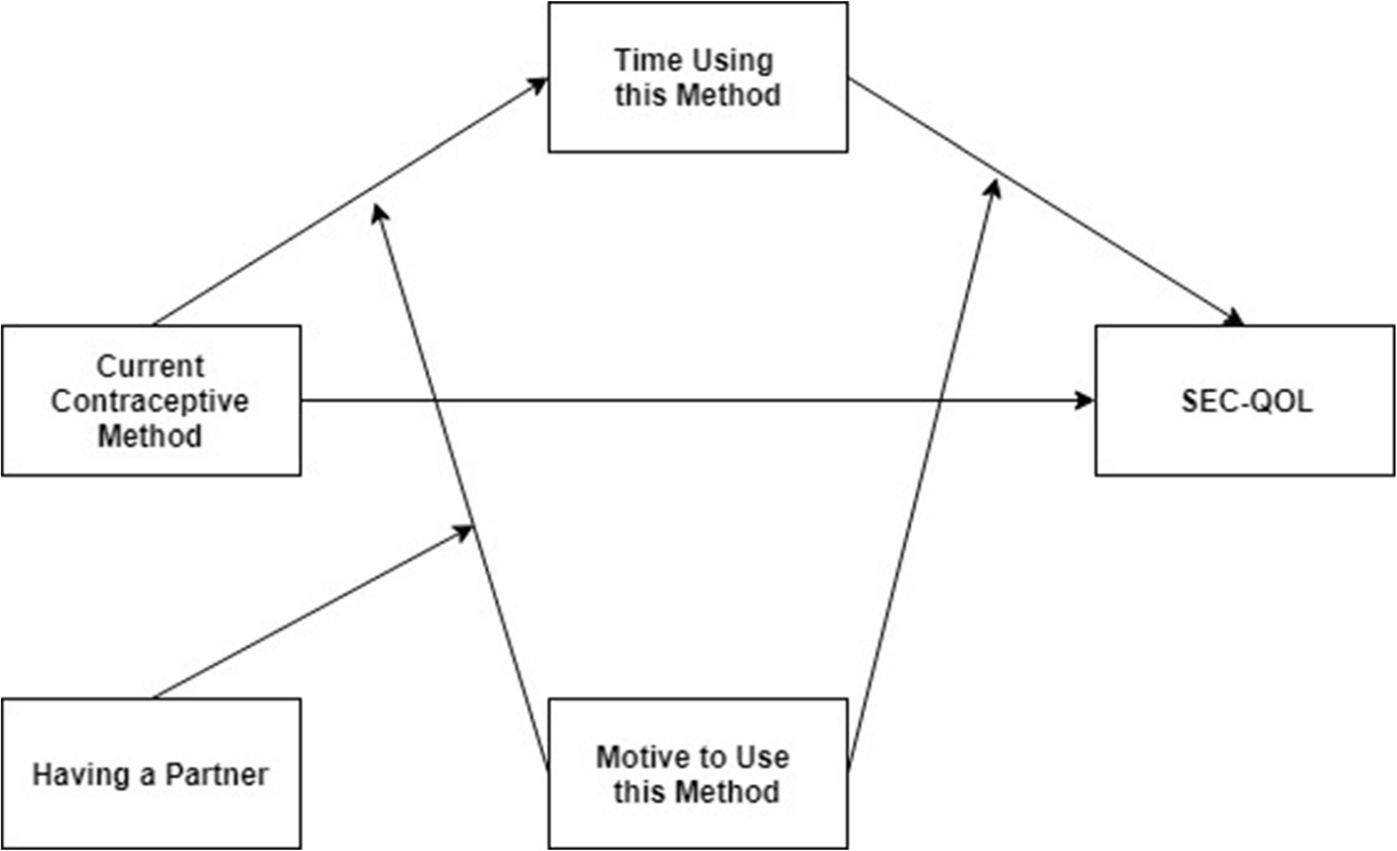
Moderated mediation model of health-related quality of life according to current contraceptive method, time using this method, motive to use it, and having a partner

First, results showed that there were differences in SEC-QoL by the contraceptive method used (β = 0.12, *p* = 0.004, 95% CI [0.04, 0.20]), so that higher scores in SEC-QoL were found in participants who used hormonal methods (M = 51.32, SD = 17.86), while lower scores were observed in those who used non-hormonal methods (M = 46.08, SD = 16.57), F(2, 861) = 8.69, *p* < 0.001, η^2^_p_ = 0.02. Second, these differences on SEC-QoL were partly related to the time using that method. Moreover, the reason for using the contraceptive method and having a partner were proposed as moderators in that partial mediation. Results showed, on the one hand, some differences in the time using the current contraceptive method according to the specific method (β = − 0.11, *p* = 0.010, 95% CI [− 0.20, − 0.03]. Consequently, participants who used non-hormonal methods reported more months using them (M = 35.42, SD = 29.74) than those who used hormonal methods (M = 28.15, SD = 25.04), which is expectable on the basis of participants’ sexual life course. Moreover, the relationship between the current contraceptive method and the time in months using that method was found to be moderated by the reason for using it and having a partner or not (β = − 0.10, *p* = 0.021, 95% CI [− 0.19, − 0.02]), F(7, 545) = 3.16, *p* = 0.003, R^2^ = 0.04. Thus, among participants who used non-hormonal contraceptive methods because of its security, those who had a partner reported more months using it (M = 36.32, SD =30.20) than those without a partner (M = 26.86, SD = 22.82). Moreover, among participants who used non-hormonal contraceptive methods because of its availability, those who had a partner reported more months using it (M = 27.11, SD = 38.77) than those without a partner (M = 18.00, SD = 8.49). On the other hand, results indicated that the motive to use the current contraceptive method also moderated the relationship between the time using it and the scores in SEC-QoL (β = 0.11, *p* = 0.004, 95% CI [0.04, 0.19]), F(3, 567) = 4.73, *p* = 0.002, R^2^ = 0.02. Among participants who had used the current contraceptive methods for more than 24 months, the motives of availability (M = 72.00, SD = 19.97) and price (M = 63.17, SD = 17.45) were associated with higher health-related quality of life compared to those participants with less than 24 months using that method (M = 53.22, SD = 9.77, and M = 56.46, SD = 14.19, respectively). Moreover, greater health-related quality of life was also found in undergraduate women that used the current contraceptive method for more than 24 months in order to prevent an STI (M = 58.76, SD = 12.83), compared to those who shared this same motive but had used the method for less time (M = 55.80, SD = 12.99). However, no significant association was observed between the time using the contraceptive method and health-related quality of life scores (β = 0.03, *p* = 0.436, 95% CI [− 0.05, 0.12]. Figure 1 presents the integration of the previous results into a single moderated mediation model. Thus, this model showed that the relationship between the current contraceptive method and the scores in overall SEC-QoL was partially associated with the moderated mediation of time in months using this method, the motive to use this method and having a partner or not, F(4, 548) = 5.81, *p* < 0.001, R^2^ = 0.04.

## Discussion

The questionnaire used in this study is specific for women using contraceptive methods to address its effect on health-related quality of life. We measured five health-related quality of life dimensions (social, menstrual symptoms, breast symptoms, psychological and sexual) that allowed us to find differences related to the use of contraceptives. There have been few studies assessing the impact of contraceptive use on women’s quality of life with such a specific questionnaire as SEC-QoL [26–28].

This study found that women who use any contraceptive method have higher scores of SEC-QoL than those who use no contraceptive. When comparing different methods of contraception, women who used hormonal methods had higher scores. Actually, women who use any form of contraception may have an improved health-related quality of life and different contraceptive methods have varying degrees of impact on women’s. However, it is difficult to determine the exact impact of contraception on health-related quality of life [32] so this is a multifactorial construct involving social and cultural factors [33].

We asked about the motivation for using contraceptives. Some may use them to prevent a pregnancy and others for non-contraceptive benefits. The main reason is to avoid unplanned pregnancies. In other studies, with young women, the main reason for choosing a method is to plan a pregnancy [34].

Women obtained more benefits other than contraceptive effects. Menstrual symptoms’ dimension of SEC-QoL was shown to be less severe in women who chose hormonal contraceptive methods. In our study, we observed an improvement in these domains of SEC-QoL in those women who chose hormonal contraceptive method: breast tenderness, social, menstrual and sexual symptoms. These results are consistent with previous studies [23, 35]. Likewise, the psychological domain was higher in the group that took oral contraceptives, but not in a statistically significant manner. This is similar to what happened in other studies where the scores of the groups that used hormonal methods were significantly improved by taking oral contraceptives [36]. According to previous studies, premenstrual breast tenderness had a great impact on the life of women who experienced it [37, 38]. In our study, women who use a hormonal contraceptive feel that breast tenderness less. When these adverse events are reduced, this contributes to improving overall health-related quality of life. However, whether the combined oral contraceptive pill reduces breast pain is not clear [37].

There is a lack of knowledge about how contraceptives affect sexual function. Zethraeus et al. showed no negative impact of oral contraceptives on overall sexual function [39]. In this line, our study showed that women who used contraceptive methods had fewer score in the dimensions of menstrual and better sexual health-related quality of life. Similar results were obtained by Caruso et al. [38]. We also observed that women using oral contraceptives had sex more frequently. On the other hand, other studies showed that combined oral contraceptives did not improve sexual life or health-related quality of life [22]. Besides, many factors may contribute to having effects on female sexuality, such as psychosocial and cultural influences [38].

This sample is formed by women in their twenties, so we do not have users of definitive methods or intrauterine dispositive, more common in older women. Most women in this study reported using hormonal methods or a condom as in others studies carried out in Spain [34, 40]. We cannot establish a relationship between those methods and health-related quality of life as it was showed in other pieces of research [26, 41] since these methods are mostly used among elder women. As a contribution of the present research, a moderated mediation model has been developed to examine the interactive effects of some variables regarding the use of contraceptive methods on the overall scores in health-related quality of life. This model showed that the current contraceptive method had a relationship with health-related quality of life both directly and through its relationships with the time using it, the motive to use it and having a partner or not. Thus, several variables should be considered in order to study the health-related quality of life of undergraduate women on the basis of their experience with contraceptive methods [42].

The amount of time using a method is not directly related to higher scores, so we could presume that positive effects are felt sooner. Students who have been using contraceptives longer did not show higher scores on health-related quality of life. Zocal’s study showed that after 6 months SEC-QoL global and dimension scores had greater increases from baseline in women who chose to switch to combined oral contraception based on natural estrogen [27]. A revision by Lopez et al. found better scores after only 3 months [1].

As a limitation of this study, we state that we did not measure the longitudinal effect of contraception on premenstrual syndrome from a baseline. We asked women about their perception of health-related quality of life at one specific point and in retrospect by applying a questionnaire. Other limitation that should be acknowledged is the need to assess other relevant variables, such as beliefs about premenstrual symptoms [43], and a further examination of all the premenstrual symptoms listed in Diagnostic and Statistical Manual of Mental Disorders – V (DSM-V). Due to possible bias toward false positive reports in retrospective self-report measures of premenstrual changes, prospective daily monitoring of all symptoms for at least two consecutive menstrual cycles may be recommended [44]. Moreover, the type and amount of hormones that women took should be controlled, and this improvement may be recommended as a future research line. Furthermore, because a cross-sectional design was followed, only associations among variables can be concluded. The moderated-mediation model based on regression analyses only represents causal assumptions. A longitudinal design is recommended to examine relationships between antecedents and consequents and an experimental design is required to test causal effects.

## Conclusions

The achieved results suggest higher scores of SEC-QoL in women using hormonal contraceptive methods. Overall, women reported better menstrual health-related quality of life, lower breast tenderness and a better sexual life.

## Appendix

### The SEC-QoL Questionnaire

This English version of the questionnaire is a simple translation of the Spanish version to show its contents.

1. I have menstrual pain (pain in the lumbar area and abdomen) a few days before my period starts.
2. I feel a mild discomfort in the kidney area a few days before my period starts.
3. I feel discomfort in the ovary area during my period.
4. My breasts feel harder during my period.
5. I feel that my breasts increase in size on the days before my period starts.
6. My breasts increase in size during my period.
7. I feel more nervous and I have less patience on the days before and during the first few days of my period.
8. My legs feel more tired on the days before and during the first few days of my period.
9. On the days before and during the first few days of my period, I do not feel like doing sport or any activity that is related to sudden movement.
10. On the days before and during the first few days of my period I prefer to be calmer and to do fewer things.
11. When my period coincides with the weekend, I refrain from doing things because of physical discomfort.
12. When I have my period I am more nervous at work.
13. My sexual desire decreases during the days of my period.
14. On the days before and during the first few days of my period, I feel nervous and susceptible (I get upset about anything).
15. I worry that I may have hormonal disorders.
16. When my period comes, my performance at work is somewhat lower because of menstrual pain.
17. I worry that the birth control method I use in my sexual relations may fail.
18. During the first few days of my period, when bleeding is heavier, I refuse sexual intercourse.
19. During my period, I feel that I have less energy (everything requires me more effort).

## Abbreviations

DSM-V: Diagnostic and Statistical Manual of Mental Disorders – V
EQ-5D: EuroQol-5D
PGWBI: Psychological General Well-Being Index
PMS: Premenstrual syndrome
Q-LES-Q: Quality of life Enjoyment and Satisfaction Questionnaire
SEC-QoL: Sociedad Española de Contracepción- Quality of Life
SPSS: Statistical Package for the Social Sciences
STI: Sexual transmission infection
